# Association of Vitamin D3 Level with Breast Cancer Risk and Prognosis in African-American and Hispanic Women

**DOI:** 10.3390/cancers9100144

**Published:** 2017-10-24

**Authors:** Yanyuan Wu, Marianna Sarkissyan, Sheilah Clayton, Rowan Chlebowski, Jaydutt V. Vadgama

**Affiliations:** 1 Division of Cancer Research and Training, Charles R. Drew University of Medicine and Science, 1731 East 120th Street, Los Angeles, CA 90059, USA; yanyuanwu@cdrewu.edu (Y.W.); mariannasarkissyan@cdrewu.edu (M.S.); sheilahclayton@cdrewu.edu (S.C.); 2 Jonsson Comprehensive Cancer Center, David Geffen School of Medicine, University of California at Los Angeles, Los Angeles, CA 90095, USA; rchlebow@whi.org; 3 Harbor UCLA Medical Center, Torrance, CA 90509, USA

**Keywords:** vitamin D, breast cancer, African-American women, Hispanic women, disparity, metastases

## Abstract

*Background*: This study investigated the association of vitamin D3 levels with breast cancer risk and progression in African-Americans and Hispanics. *Methods*: A total of 237 African-American (Cases = 119, Control = 118) and 423 Hispanic women (Cases = 124, Control = 299) were recruited in the study. Blood samples were collected at the time of breast cancer screening and prior to cancer treatment for 4 weeks on average for the cases. The serum 25-hydroxyvitamin D (25(OH)D3) was measured at a Quest-Diagnostics™ facility. *Results*: The results showed that 69.2% of African-Americans and 37.8% of Hispanics had 25(OH)D3 levels below 20 ng/mL. The 25(OH)D3 level below 20 ng/mL was significantly associated with breast cancer in both African-Americans (OR = 2.5, 95% CI = 1.3–4.8) and Hispanics (OR = 1.9, 95% CI = 1.1–3.0). However, the predicted probabilities of breast cancer in African-Americans were significantly higher than in Hispanics (*p* < 0.001). The 25(OH)D3 below 20 ng/mL was significantly associated with triple negative breast cancer (TNBC) in African-Americans (OR = 5.4, *p* = 0.02, 95% CI = 1.4–15), but not in Hispanics in our cohort of participants. Levels of 25(OH)D3 below 26 ng/mL predicts a decrease in disease-free survival, but it was not an independent predictor. *Conclusions*: Our data shows an association between 25(OH)D3 levels and the risk of breast cancer. Further studies on the relationship between 25(OH)D3 level and breast cancer risk are warranted.

## 1. Introduction

Breast cancer is the most common cancer and the second leading cause of cancer death in women in the US. African-American women with breast cancer have the highest mortality rates compared to other ethnic groups and for all age groups [1]. Laboratory and animal studies show that vitamin D may affect cancer cell growth, apoptosis, and tumor angiogenesis at cellular level [2,3,4,5]. A number of epidemiologic studies have suggested a positive association between higher vitamin D intake or vitamin D higher blood levels and lower incidence of breast cancer [6,7,8,9,10,11,12]. However, many studies have shown inconsistent trend between vitamin D levels and risk of breast cancer. Some studies supported the hypothesis that high circulating vitamin D level reduces the risk of breast cancer [8,9,10] and others did not [6,7]. A pooled analysis from 11 case-control studies supported the hypothesis that higher serum 25(OH)D levels reduce the risk of breast cancer [13], while another meta-analysis on 24 studies (10 studies on vitamin D intake and 14 studies on serum 25(OH)D levels) concluded that high 25(OH)D was weakly associated with low risk of breast cancer, but strongly associated with better breast cancer survival [14]. The different study designs could be one of main reason for the inconsistent results. Case-control studies were more likely to provide reliable results on the effect of vitamin D for reducing risk of breast cancer [15].

Most of these studies reported data on Caucasians. Only few of these studies were on Hispanic/Latino populations, and the results were contradictory [10,11,12]. Even though a few studies included a comparison between Caucasians and African-Americans, the determination of odds ratio (OR) or relative risk (RR) was not ethnic specific [6,16]. An earlier study on a multi-ethnic cohort of breast cancer cases suggested an association of lower 25(OH)D levels with breast cancer and with negative estrogen and progesterone receptors (ER/PR) [17], but another study [18] did not support this finding. The reports on the association of 25(OH)D level and breast cancer patients’ survival were also inconsistent [17,18]. A previous study has suggested that 25(OH)D concertation inversely related with incidence and/or mortality rates for many cancers including breast cancer and the differences in the vitamin D status may account for disparities in cancer survival between African-Americans and Caucasians [19]. Recently a meta-analysis suggested an inverse relationship between the 25(OH)D level and overall survival in breast cancer patients, however, the cut-off level for deficiency was variated by studies in this analysis [20]. A prospective cohort study of breast cancer survivors from Kaiser Permanente Northern California (Oakland, CA, USA) identified that 25(OH)D serum levels were independently associated with breast cancer prognosis in premenopausal women only [21]. There is a significant lack of studies investigating the association between 25(OH)D level and survival in African-American and/or Hispanic breast cancer patients. Some studies have reported either significant or non-significant association of 25(OH)D level with ER/PR status in breast cancer. Less is known on its association with other subtypes of breast cancer such as HER2 and Triple Negative breast cancers.

It has been reported that African-Americans and Hispanic-Americans tend to have lower circulating vitamin D levels compared to Caucasians [22]. Hence it is important to assess the role of circulating vitamin D levels in breast cancer in these women, specifically the association between low vitamin D levels with breast cancer subtypes. Thus, our current study aims to assess the association of circulating vitamin D levels with: (a) breast cancer; (b) clinicopathological breast cancer features; (c) breast cancer disease-outcome in a hospital-based setting within a community comprised mostly of self-identified African-American and Hispanic individuals.

## 2. Results

A total of 660 women participated in our study, divided into two groups: “case” and “control”. The case group had 243 (36.8%) women diagnosed with breast cancer and the control group had 417 (63.1%) women who did not have cancer. The self-identified ethnic distribution of the subjects is shown in Table 1. A total of 237 (35.9%) were African-American, with 119 (50.2%) in case group and 118 (49.8%) in the control group. A total of 423 were Hispanic women, with 124 (29.3%) in case group and 299 (70.7%) in the control group. The age range of women included in this study was between 21 –80 years old. The median age of the case group was 51 years old, ranged from 28–80, and the median age of the control group was 48 years, ranged from 20–79. Table 1 shows the age distribution by percentiles between case and control groups in African-Americans and Hispanics. No significant difference was found in age between case and control groups in African-American women. The median age of the control was younger than the median age of case group in the Hispanic cohort (*p* = 0.005). Overall, the age of Hispanic women was younger than African-American women in this study. As shown in Table 1, the majority of women in this cohort are in the obese range (Body Mass Index, BMI ≥ 30).

### 2.1. 25(OH)D3 Level and Ethnicity

**Figure 1:**
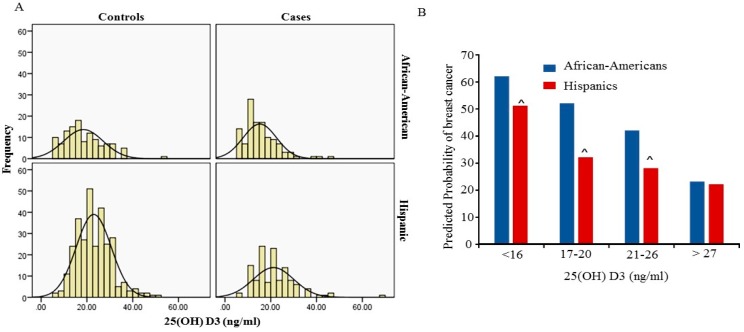
Serum 25 (OH)D3 level and its association with breast cancer in African-American and Hispanic women. (**A**) the bar graphs demonstrates the distribution of serum 25(OH)D3 levels between case and control groups in African-Americans and Hispanics as indicated; (**B**) Probability of breast cancer by serum 25(OH)D3 in different percentiles was predicted by Logistic regression utilizing IBM SPSS version 22, blue bar indicates the probability in African-Americans and red bar indicates the probability in Hispanics. ^ *p* < 0.05 indicates the probability of breast cancer compared to African-Americans in the indicated range of 25(OH)D3 level.

The mean level of 25(OH)D3 and the frequency of 25(OH)D3 deficiency (<20 ng/mL) between African-American and Hispanic women are shown in Table 2 and Table 3. Overall the 25(OH)D3 level in African-American women was significantly lower than in Hispanic women (Table 2). The level of 25(OH)D3 was also significantly lower in breast cancer cases than in controls in both African-American and Hispanic cohorts. There were no significant difference in 25(OH)D3 level among age groups in both African-Americans and Hispanics. The lowest level of 25(OH)D3 was observed in age group between 31 to 50 years in African-American women. Figure 1A shows the distribution of 25 (OH)D3 level between cases and controls in African-Americans and Hispanics. The peaks of the distribution curve were in the deficiency range (<20 ng/mL) for African-Americans and in the insufficiency (>20 ng/mL and <30 ng/mL) for Hispanics respectively. Overall a total 69.2% of African-American women had 25(OH)D3 levels below 20 ng/mL and 37.8% of Hispanic women had 25(OH)D3 levels below 20 ng/mL. The frequency of 25(OH)D3 levels below 20 ng/mL was significantly higher in African-Americans compared to Hispanics (*p* < 0.001). In this study we did not find an association between the level of 25(OH)D3 and BMI in both African-Americans and Hispanics.

### 2.2. Association of 25(OH)D3 Level and Breast Cancer

Utilizing univariate analysis we were able to demonstrate that there was a significant association between 25(OH)D3 deficiency and breast cancer in the total cohort (OR = 2.2; 95% CI = 1.6–3.0; *p*-value < 0.001) (Table 4). Furthermore, when evaluating the association of 25(OH)D3 level with breast cancer by ethnicity, a significant association of 25(OH)D3 level with breast cancer was found in African-Americans (OR = 2.4; 95% CI = 1.3–4.2; *p*-value = 0.003) and Hispanics (OR = 1.5; 95% CI = 1.0–2.4; *p*-value = 0.05). A linearized inverse association between the predicted probabilities of breast cancer with the serum 25(OH)D3 levels was seen in both African-Americans (R = 0.934, R^2^ = 0.872, *p* < 0.001) and Hispanics (R = 0.8, R^2^ = 0.6, *p* < 0.001). However, the predicted probability of breast cancer increased rapidly in African-Americans than in Hispanics at each percentile (*p* < 0.001) (Figure 1B). As shown in Figure 1B, when serum 25(OH)D3 is in the range of insufficient levels (20–26 ng/mL) the predicted probability of breast cancer in African American women is 43% and 27% in Hispanic. However, the probability of breast cancer increased to more than 52% in African-Americans and more than 31% in Hispanics who had serum 25(OH)D3 deficiency (less than 20 ng) respectively. Since age and BMI are known to be associated with breast cancer, multivariable analysis, adjusted for age and BMI, was also performed. The multivariate analysis for the total cohort was performed with adjusted ethnicity, age and BMI, and confirmed the relation between deficiency in 25(OH)D3 levels with breast cancer (OR = 2.0; 95% CI = 1.4–3.0; *p*-value = 0.001). Furthermore, the significant association of 25(OH)D3 deficiency with breast cancer was confirmed by multivariate analysis in both African-Americans (OR = 2.5; 95% CI = 1.3–4.8; *p*-value = 0.01) and Hispanics (OR = 1.9; 95% CI = 1.1–3.0; *p*-value = 0.01).

### 2.3. Serum 25(OH)D3 Level in Breast Cancer Patients

In Table 5, the clinicopathological features of breast cancer were assessed to determine association with 25(OH)D3 levels. The data showed that African-American women with triple negative breast cancer (TNBC) had the lowest 25(OH)D3 levels. Logistic regression with multivariate analysis adjusted for BMI, age at the time of diagnosis and seasons of blood draw, showed that the serum 25(OH)D3 level below 20 ng/mL had a significant association with TNBC type of tumor in African-American women (OR = 5.4, *p* = 0.02) (Table 6).

The level of 25(OH)D3 had no association with either ER/PR+/HER2− tumor or ER/PR−/HER2− tumor. There were no significant differences in the 25(OH)D3 levels among the different ER/PR, HER2 status, tumor size, lymph node involvement, and tumor stages in both African-Americans and Hispanics groups (Table 5). As shown in Table 5 African-American women with breast cancer overall had significantly lower levels of 25(OH)D3.

The Cox regression with univariate and multivariate analysis adjusted for ethnicity, ER/PR/HER2 receptors, tumor size, node involvement, BMI, age at the time of diagnosis and the seasons of blood draw were performed to assess the relative risk (RR) for reducing disease-free survival (DFS) and overall survival (OS).

The relative risk (RR) for DFS increases with decrease in the levels of 25(OH)D3 per quartile. Univariate analysis (Table 7) showed that patients who had 25(OH)D3 level between 13 ng/mL and 23 ng/mL had 2.3-fold risk of cancer relapse or metastases compared to patients with the level above 23 ng/mL (*p* ≤ 0.03). Patients with 25(OH)D3 level ≤ 12 ng/mL also increase RR to 1.7, however, it was not statistically significant and this may be due to less number of patients in this quartile. Similar trend was seen in RR for overall survival (OS) (Table 7). Multivariate analysis in Table 7 showed that the RR for reducing DFS still remained as 2.2 to 1.9 when the 25(OH)D3 levels were below 20 ng/mL, and the RR for reducing OS was also increased per quartile decreasing the level of 25(OH)D3 (Table 7). However, when the analysis performed by stratifying ethnicity no significant association was found between the level of 25(OH)D3 level and the disease-outcome (DFS and OS) in either African-Americans or Hispanics.

## 3. Discussion

The vitamin D deficiency has been defined as a serum 25 (OH)D3 level less than 20 ng/mL and the 25(OH)D3 level of 21 ng/mL to 29 ng/mL is considered insufficiency for vitamin D [22]. Based on this definition 41.6% of US adults have vitamin D deficiency [23]. It has been reported that sufficient vitamin D levels decrease the risk of breast cancer to up to 45% [24]. African-Americans and Hispanics are more likely to suffer from vitamin D deficiency compared to Caucasians [25]. The vitamin D deficiency has been reported to be associated with poor/fair health status and significantly associated with obesity, hypertension and lower HDL cholesterol levels [23], while the association of vitamin D level with breast cancer was uncertain. Case-control studies from different populations showed that serum 25(OH)D3 level was associated with breast cancer significantly. A case-control study in Mexican women showed a significant inversed association between serum 25(OH)D3 levels and breast cancer in both pre- and postmenopausal women [10]. Similar results were found in a case-control study with Korean women [11]. A study from Germany reported that the low serum 25(OH)D3 level was associated with risk of postmenopausal breast cancer only [9]. However, results from a study in the Cancer Prevention Study-II Nutrition Cohort do not support relationship between adulthood serum 25(OH)D and postmenopausal breast cancer [6]. The inconsistency in the results could be due to the heterogeneity in the study population, time of sample collection, methods in determination of the level of serum vitamin D.

In this study we examined the association between serum 25(OH)D3 levels and breast cancer using pre-cancer treatment serum from African-American and Hispanic women patients at the breast clinic at the Martin Luther King (MLK) Medical Center in South Los Angeles (CA, USA). Both the African-American and Hispanic women in this study have similar socio-economic status and access to health care. In our study cohort Hispanic women were self-reported mostly of Mexican-Hispanic origin. Our data showed that both African-American and Hispanic women had low serum 25(OH)D3 levels. The mean levels of 25(OH)D3 were in the range of insufficiency for the Hispanic women and in the range of deficiency for the African-Americans in both groups “case” and “control”. This trend was consistent with the data from National Health and Nutrition Examination Survey (NHANES) [25]. We observed 25(OH)D3 deficiency in the younger African-American women in the cohort. The deficiency of 25(OH) D3 was seen in both African-American cases and controls above 30 years old. Overall the serum 25(OH)D3 level is significantly lower in African-American than in Hispanic women. The lowest level of 25(OH)D3 was found to be in age between 31 and 50. The data from this study supports the association of serum vitamin D levels and risk of breast cancer. We found a significant association between low serum 25(OH)D3 levels and breast cancer in both African-American and Hispanic women. Interestingly our data showed that the predicted probability of breast cancer was 32% to 42% in African-American women and 25% to 27% in Hispanic women who have 25(OH)D3 insufficiency (21–30 ng/mL). The predicted probability of breast cancer increases to 52% in African-American and 32% in Hispanic women when the 25(OH)D3 level decreases to 12–19 ng/mL. The participation rates for the “case” group is 89% (330/368) and 63.4% (610/962) for the “control “group in this study. The lower participation rates in the control group is mainly due to unclear documentation on date of birth and breast cancer screening results in medical records, small percentage (less than 10%) is due to missing blood samples. The most of those missing control participants were Hispanic women. Since we have more controls from Hispanics in our study we don’t anticipate non-response bias for our results. However, the significant difference in the predicted probability could be influenced by some confounding factors existing between African-Americans and Hispanics, such as meat consumption [26,27]. Meat is an important source of Vitamin D and also may associate with breast cancer if it is prepared in certain ways [28,29]. Further study is required to confirm our observations and to understand the insight mechanisms of vitamin D affecting the risk of breast cancer in African-American women.

Vitamin D is a steroid hormone and the mechanisms underlying for vitamin D in cancer development are not well understood. Some evidence suggest that vitamin D is involved in the regulation of cell growth, apoptosis and cell differentiation [30,31,32,33]. Its biological activities can be reached through binding to a specific high-affinity receptor, vitamin D receptor (VDR) [34] or being independent of VDR-pathway [35]. The CYP27B1 and CYP24A1, the main enzymes involved in vitamin D metabolism, also regulate the biological activities of vitamin D [36]. Besides, mutant p53 was reported to be able to modulate cells response to vitamin D3. The mutation in p53 could influence vitamin D3-induced transcription and converts vitamin D3 from a pro-apoptotic into an anti-apoptotic effector [37]. It has been well known that the mutations in the p53 gene is a frequent event in human cancer [38,39]. Alteration in p53 was found more common in African-American women than that in Caucasian women with breast cancer [40,41]. Whether the high frequency of alternation in p53 contributes to the high probability of breast cancer need to be investigated in future.

Several studies have shown that the low level of vitamin D was more likely to be associated with women with obesity [8,9,23,42,43]. However, we did not find any significant association between serum 25(OH)D3 levels with either obese or overweight subjects from our study. This could be due to (a) the majority of subjects in this study were either overweight or obese; and (b) most of the women in the study has either deficient or insufficient serum 25(OH)D3 levels.

It has been reported that vitamin D deficiency is inversely associated with histological grade and tumor stage in breast cancer [44,45]. The data from our study did not support these associations. We did not observe significant association of 25(OH)D3 level with breast tumor stage in both African-American and Hispanic patients. A significant association of serum 25(OH)D3 below 20 ng/mL with TNBC was observed in African-American patients in this study, but not in Hispanic patients. The reason for not observing a clear association in Hispanic patients may be due to small sample size. Further studies with larger sample size are warranted. No association of 25(OH)D3 level with other subtypes of tumor was found in both African-Americans and Hispanics in this study. Similar to our findings, a study from Caucasian and non-Caucasian (undefined ethnic groups) subjects revealed that breast cancer patients with TNBC had lower 25(OH)D3 level [46]. Another case-control study from Caucasian subjects demonstrated the significant association of low 25(OH)D3 with ER-negative or TNBC in premenopausal women only [47]. A prospective cohort study of breast cancer survivors from Kaiser Permanente Northern California showed the association of lower serum 25(OH)D concentrations with advanced-stage tumors, and TNBC in premenopausal women [21]. Their study also identified that serum 25OHD levels were independently associated with breast cancer prognosis in premenopausal women. While some studies failed to show this relationship [44,48,49]. The classification of ER status and/or assessment of 25(OH)D3 level (dietary, supplement taken and the time of blood samples collection etc.) may contribute to the discrepancy in different studies. In addition, genetic variants in the vitamin D pathway, such as VDR and CYP24A1 have been shown to have influence in serum 25(OH)D3 levels and be more related to ER-negative tumor [50]. These Single Nucleotide Polymorphisms (SNPs) have been found to be different between African-Americans and European-Americans in the same study. The increased risk of ER-negative breast cancer with low serum 25(OH)D3 levels in African-American compared to European-American was reduced and become non-significant after adjusting for the SNPs [44]. Our previous studies in VDR SNPs from the same cohort of African-American and Hispanic women showed that specific VDR gene polymorphism was associated with breast cancer in African-Americans and predict DFS only [51]. Further studies on the association of VDR SNPs with the levels of 25(OH)D3 and risk of TNBC in African-American and Hispanic women are ongoing in our Laboratory. We are confident that the outcome of these studies will elucidate further mechanisms that will provide a better understanding for the association of vitamin D levels with breast cancer subtypes.

Our study is one of the few studies that compares the association between serum 25(OH)D3 level and incidence of breast cancer between African-American and Hispanic living in south Los Angeles with similar lower social-economic status. South Los Angeles and/or SPA6 area had 31% of population that lives in poverty (household income < 100% federal poverty level (FPL)) and median household income is $36,400 (Los Angeles county is 17% FPL and median household is $56,241) [52]. The findings from this study add to our current knowledge of serum 25(OH)D3 and breast cancer in minority and underserved populations. Since we have the follow-up information on our breast cancer patients, it allows us to analyze the RR of DFS and OS by the deficiency in serum 25(OH)D3 in African-American and Hispanic women. A more than 2.3-fold increase in RR of reducing DFS was seen in African-American and Hispanic patients with serum 25(OH)D3 between 13 ng/mL to 26 ng/mL (*p* < 0.03). After adjustment for ethnicity, tumor size, node stage, ER/PR/HER2 status, seasons of blood draw, BMI and age at the time of diagnosis the RR of reducing DFS was still remaining at 1.9 to 2.2 when the level of 25(OH)D3 below 20 ng/mL. The RR for reducing OS was also increased per quartile with decreasing level of 25(OH)D3 in our study. However, once the analysis was performed by stratifying ethnicity the RR lost statistical significance in both univariate and multivariate analyses. In this study we were not able to adjust other potential confounders, such as alcohol usage, smoking, oral contraceptives using and physical activities since this information was not collected for all of subjects. Further study with an enlarged sample size or by adjustment for all of those potential confounders need to be conducted to verify the significant association between the level of 25(OH)D3 and the disease-outcome of breast cancer in African-American and Hispanics.

In summary, data from this study showed a significant inverse association between serum 25(OH)D3 levels and breast cancer risk in African-American and Hispanic women. The predicted probability of breast cancer was significantly higher in African-American women with 25(OH)D3 level below to 26 ng/mL compared to that in Hispanic women with same level of 25(OH)D3. A significant association of 25(OH)D3 level (below 20 ng/mL) with TNBC tumor was seen in African-American patients. The 25(OH)D3 level below 13 ng/mL predicts reduced DFS, but it was not an independent predictor. The significance was lost after adjustment for tumor characteristics, age, BMI and the seasons of blood draw. The data suggests that the benefits of vitamin D may be included in reducing the risk of breast cancer and perhaps in reducing the risk of TNBC. Further studies are also warranted to understand the influence of SNPs in the vitamin D induced VDR pathway. Assessment of serum vitamin D levels could provide a positive benefit toward identification of at risk populations for breast cancer, and subsequently provide better intervention strategies for prevention of breast cancer.

## 4. Methods and Materials

### 4.1. Subjects

The study population was recruited from SPA6 region of South Los Angeles County in California. The population by race/ethnicity in SPA6 are 28% African-American, 68% Hispanic/Latino, and 4% others that include Caucasian, Asian, Native-American and Pacific-islander. The cohort comprised of women examined in the Mammography Clinic or the Hematology/Oncology Clinic at the Martin Luther King Ambulatory Care Center (MACC, formerly known as King-Drew Medical Center) between 1995 and 2007. Women were consented for an ongoing breast cancer study conducted in the Division of Cancer Research and Training at Charles R. Drew University of Medicine and Science and MACC. The study was approved by the Institutional Review Board. For follow up data, we conducted post-hoc medical records abstraction.

**Figure 2:**
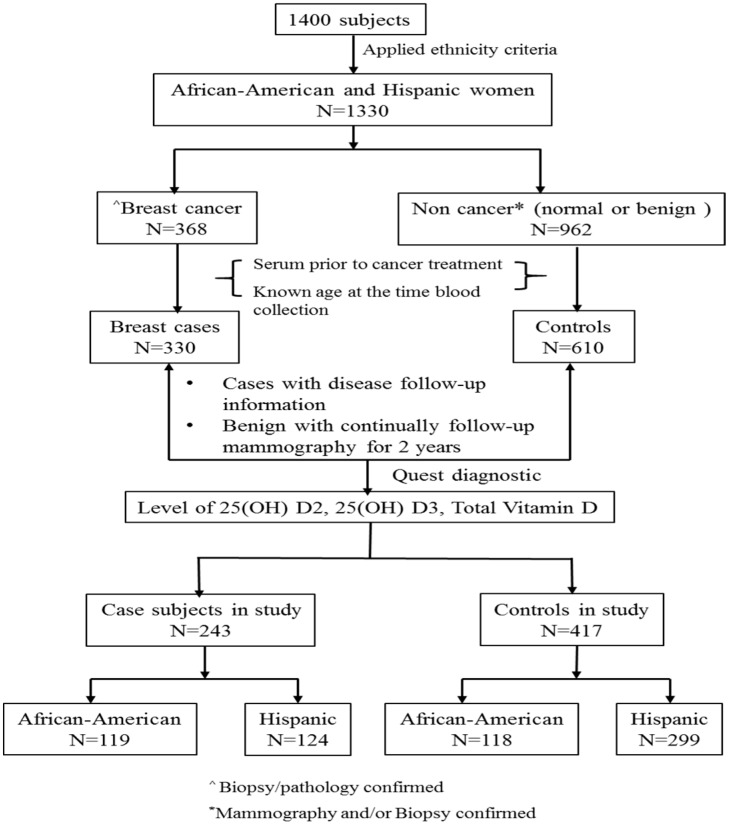
Subjects selection. The flowchart demonstrated subjects’ selection for the study. ^ All breast cancer cases had documented biopsy/pathology reports, and * all non-breast cancer subjects had documented mammography and/or biopsy.

Figure 2 summarized the process of selecting the subset of subjects for this study from total number of women (*n* = 1400). Those women were consented for the breast cancer study. The inclusion/exclusion criteria were the following: (a) Self-identified race/ethnicity: from self-report, 30% were African-American, 65% were Hispanic, and the remaining 5% were Caucasian or Asian subjects. Considering the subjects from Caucasian and Asian were relative small that will not generate meaningful analysis we only included African-American and Hispanic women in this study. When the African-American and Hispanic ethnicity criteria were applied, *n* = 1330 women met the criteria; (b) Confirmation of breast cancer: For cases, breast cancer status was determined by biopsy/pathology confirmed neoplasm of the breast, and only subjects who had documentation of this information were included in the study (*n* = 368). For controls, normal was determined by normal mammogram results, and benign was determined by mammogram and biopsy confirmed non-cancer (i.e., benign breast disease) (*n* = 962); (c) Baseline blood sample (serum sample collected at the time of diagnosis and prior to any cancer treatment for cases), documentation of age at the time of sample collection. A total 940 samples were available (330 cases and 610 controls); We selected (d) cases with documented disease follow-up information and controls with benign disease having 2-years follow-up mammography information for assessing level of 25(OH)D2, 25(OH)D3 and total vitamin D. (cases = 243 and controls = 417; total *n* = 660).

### 4.2. Serum 25-Hydroxyvitamin D Level

To measure circulating 25-hydroxyvitamin D levels a total 150 µL of serum samples prior to any cancer treatment (average 4 weeks prior treatment) was sent to a Quest-Diagnostics™ laboratory. The serum 25(OH)D2, 25(OH)D3 and total level of 25(OH)D were measured using a Liquid Chromatography/Tandem Mass Spectrometry (LC/MS/MS) method. The 25(OH)D3 indicates both endogenous production and supplementation. The 25(OH)D2 is an indicator of exogenous sources such as diet or supplementation. The total vitamin D is a combination of 25(OH)D2 and 25(OH)D3. The level of 25(OH)D2 was lower than 4 ng/mL (out of range for determination) in 88% of the subjects from our cohort. To obtain the meaningful statistical analysis the level of 25(OH)D3 was used for the study analysis. The level of vitamin D was considered “deficiency” if the 25(OH)D3 level < 20 ng/mL, “insufficiency” if the 25(OH)D3 level at range from 20 ng/mL to 29 ng/mL, or sufficiency if otherwise for this study. The 25(OH)D3 levels were also calculated according to the quantiles (25-quantile < 12 ng/mL, 50-quantile = 19 ng/mL, 75-quantile = 26 ng/mL) for assessing predicted probability of breast cancer and for assessing RR of reducing DFS and OS for breast cancer patients.

### 4.3. Clinical and Demographic Information

The information on ethnicity was based on self-report and age at the diagnosis/recruitment, Body Mass Index (BMI) and clinical information were obtained through medical chart extraction. Estrogen and progestogen receptors (ER/PR) status was considered “Positive” if > 5% of tumor cell nuclei immunoreactive, or “Negative” if otherwise. HER2 (HER2/nu) status was considered “Positive” if HER2 = 3+, “Negative” if HER2 = 0, 1+, 2+, determined by immunohistochemistry or assigned using in situ hybridization to assess HER2 gene amplification. *Tumor* size, lymph node status and TNM staging were all determined according to AJCC definitions. Tumor subtype was categorized according to the status of receptors and as follows: *(i)* ER/PR+/HER2−; *(ii)* HER2+ (ER/PR− or ER/PR+); *(iii)* Triple negative (ER/PR−/HER2−).

### 4.4. DFS and OS

Survival and disease outcome in the study were assessed by 5 year DFS and 5 year OS. DFS was assessed based on screening tests such as mammography, CT scan, Ultrasounds, Bone-scans that the patient underwent after treatment and resolution of the primary cancer. DFS for a patient was defined as not having any of the following: (1) Reoccurrence; (2) Metastatic disease; (3) New primary tumor formation at another organ site. OS was calculated from the time of diagnosis to death from breast cancer.

### 4.5. Statistical Analysis

Statistical analysis was performed using SPSS software (IBM SPSS Statistics version 22, SPSS, Inc., Chicago, IL, USA). The level of 25(OH)D3 was analyzed as both continuous variable (median and mean ± SD) and categorical variable (<20 ng/mL or ≥20 ng/mL). The statistical differences of 25(OH)D3 level among different ethnic, age group (categorised as 10 years group), BMI (categorised as obese: BMI ≥ 30, Overweight: BMI: 26–29, normal: BMI ≤ 25, no subjects’ BMI < 18.5 in this cohort). The statistical significance was evaluated by using ANOVA for mean, Mann-Whitney U test for median age and median BMI. The associations of the deficiency of 25(OH)D3 with ethnicity, age group and BMI were assessed throughout using the χ^2^-test and the p-values from χ^2^-test were adjusted with Bonferroni correction. Logistic regression with univariate and multivariate analysis were used to assess the association of deficiency in 25(OH)D3 level with breast cancer in the total and ethnically sub-categorized cohort. The multivariate analysis for the association between deficiency of 25(OH)D3 and breast cancer in the total cohort was adjusted for age, ethnicity, BMI and the seasons of blood draw. The association for ethnically sub-categorized cohort was adjusted for age, BMI and the seasons of blood draw. The level of 25(OH)D3 was also categorized by percentiles based on the distribution of 25(OH)D3 in controls. The probability of breast cancer in African-American and Hispanic was predicted by Logistic Regression according to the percentiles of 25(OH)D3 and the linear trends of the predicted probability was estimated using Regression Curve estimation. The significant differences of the trends of predicted probability between African-American and Hispanic was determined by Wilcoxon signed-rank test.

The associations of 25(OH)D3 levels with breast cancer subtypes were also determined by Logistic regression with multivariate analysis adjusted for age, BMI and the seasons of blood draw. Relative Risk (RR) of 25(OH)D3 levels for reducing DFS and OS was assessed by Cox regression with multivariate analysis adjusted for ER/PR/HER2 status, tumor size, node involvement, tumor stages, BMI, the seasons of blood draw and the age at the time of diagnosis. The 25(OH)D3 levels were categorized by quartiles based on the distribution of 25(OH)D3 level in cases and the RRs were evaluated with the level of 25(OH)D3 per quartile. Throughout all analyses, only the *p* < 0.05 was considered statistically significant.

## 5. Conclusions

Data from our study supports the hypothesis that the 25(OH)D3 level below 20 ng/mL was significantly associated with risk of breast cancer. The level of 25(OH)D3 below 26 ng/mL predicts a decrease in disease-free survival, but it was not an independent predictor. The level of 25(OH)D3 below 20 ng/mL was fund to be associated with TNBC in African-Americans. Further studies on the relationship between 25(OH)D3 level and breast cancer risk are warranted.

## Figures and Tables

**Table 1:** Characteristics of Study population.

Variables	African Americans			Hispanic		
	Cases (*n* = 119)	Controls (*n* = 118)	*p*	Cases (*n* = 124)	Controls (*n* = 299)	*p*
	Median (Range)	Media (Range)		Median (Range)	Media (Range)	
**Age (years)**	^‡^ 52 (24–79)	^‡^ 55 (32–80)	0.09	^ 49 (28–80)	^‡^ 46 (20–77)	0.005
*Percentiles*						
25	46	48		42	38	
50	52	55		49	46	
75	57	61		55	53	
**BMI** ^ψ^	* 32 (20–51)	^$^ 31 (20–52)	0.6	* 31 (20–49)	^$^ 30 (20–53)	0.19
*Percentiles*						
25	28	28		27	26	
50	32	31		31	30	
75	39	37		36	34	
	N (%)	N (%)		N (%)	N (%)	
Obesity	75 (67.0)	48 (64.8)		61 (52.6)	108 (49.3)	
Overweight	18 (16.1)	19 (25.7)		38 (32.8)	71 (32.4)	
Normal	19 (16.9)	7 (9.5)	0.62	17 (14.7)	40 (18.3)	0.31

^ *p* = 0.005; ^‡^ *p* = 0.05; * *p* < 0.001; ^$^ *p* = 0.005; ^ψ^ Body Mass Index.

**Table 2:** 25(OH)D3 level by Ethnicity.

Variables		African Americans		Hispanics	*p-Value*
	N	25(OH)D3 (ng/mL) (Mean ± SD)	N	25(OH)D3 (ng/mL) (Mean ± SD)	* (AA vs. *Hisp)*
**Breast Cancer**					
Cases	119	15.2 ± 7.3	124	21.2 ± 8.8	*<0.001*
Controls	118	18.5 ± 8.6	299	23.0 ± 7.6	*<0.001*
*p-value*		*p = 0.002*		*p = 0.04*	
**Age (years)**					
≤30	3	23.6 ± 9.5	32	23.1 ± 8.8	*0.91*
31–40	19	**15.3 ^ ± 5.7**	84	23.3 ± 8.9	*<0.001*
41–50	69	**15.8 ^ ± 7.0**	157	21.4 ± 6.7	*<0.001*
51–60	96	17.5 ± 8.9	112	22.8 ± 8.9	*<0.001*
>60	50	17.1 ± 8.5	38	22.6 ± 6.7	*≤0.01*
*p-value*		*0.51*		*0.76*	
**BMI**					
Obesity	123	16.9 ± 8.7	169	22.3 ± 7.8	*<0.001*
Overweight	37	16.6 ± 7.1	109	22.9 ± 8.5	*<0.001*
Normal	26	15.8 ± 6.5	57	21.9 ± 8.3	*0.002*
*p-value*		*0.51*		*0.63*	

^ *p* < 0.05 compared with age≤30 year; * AA: African-American, Hisp: Hispanic.

**Table 3:** 25(OH)D3 level by cancer, age and obesity between African Americans and Hispanics.

Variables	African-Americans			Hispanics			*p-Value*
	Serum 25(OH)D3			Serum 25(OH)D3			
	N	<20 ng/mL N (%)	≥20 ng/mL N (%)	N	<20 ng/mL N (%)	≥20 ng/mL N (%)	*(AA* vs. *Hisp)*
**Breast Cancer**							
Cases	119	93 (78.2)	26 (21.8)	124	56 (45.2)	68 (54.8)	*<0.001*
Controls	118	71 (60.2)	47 (39.8)	299	104 (34.8)	195 (65.2)	*<0.001*
*p-Value*	*0.002*			*0.04*			
**Age (years)**							
≤30	3	1 (33.3)	2 (66.7)	32	12 (37.5)	20 (62.5)	*n.s.*
31–40	19	16 (84.2)	3 (15.8)	84	27 (32.1)	57 (67.9)	*<0.001*
41–50	69	45 (65.2)	24 (34.8)	157	64 (40.8)	93 (59.2)	*<0.001*
51–60	96	68 (70.8)	28 (29.2)	112	42 (37.5)	70 (62.5)	*<0.001*
>60	50	34 (68.0)	16 (32.0)	38	15 (39.5)	23 (60.5)	*0.02*
*p-value*	*0.51*			*0.75*			
**BMI**							
Obesity	123	84 (68.3)	39 (31.7)	169	67 (39.6)	102 (60.4)	*<0.001*
Overweight	37	26 (70.3)	11 (29.7)	109	36 (33.0)	73 (67.0)	*<0.001*
Normal	26	20 (76.9)	6 (23.1)	57	22 (38.6)	35 (61.4)	*0.002*
*p-value*	*0.51*			*0.91*			

AA: African-American, Hisp: Hispanic.

**Table 4:** Association of 25(OH)D3 and breast cancer.

Serum 25(OH)D3	Univariate Analysis			Multivariate Analysis		
	OR ^§^	95% CI	*p-Value*	OR	95% CI	*p-Value*
**Total**						
>20 ng/mL vs. ≤20 ng/mL	2.2	1.6–3.0	*<0.001*	2.0 *	1.4–3.0	*0.001*
**African-Americans**						
>20 ng/mL vs. ≤20 ng/mL	2.4	1.3–4.2	*0.003*	2.5 ^^^	1.3–4.8	*0.01*
**Hispanics**						
>20 ng/mL vs. ≤20 ng/mL	1.5	1.0–2.4	*0.05*	1.9 ^^^	1.1–3.0	*0.01*

^§^ Odds Ratio; * adjusted for ethnicity, age, BMI and the seasons of blood draw; ^ adjusted for Age, BMI and the seasons of blood draw.

**Table 5:** 25(OH)D3 level in breast cancer patients.

Variables	Total Subjects		African-Americans		Hispanics		*p-Value*
	N	25OHD3	N	25OHD3	N	25OHD3	
		Mean ± SD		Mean ± SD		Mean ± SD	
**ER/PR**							
Positive	118	18.0 ± 8.3	60	15.4 ± 8.1	58	20.7 ± 7.6	*<0.001*
Negative	99	18.1 ± 8.1	50	14.9 ± 6.2	49	21.3 ± 8.4	*<0.001*
*p-Value*	*n.s.*		*n.s.*		*n.s.*		
**HER2**							
Positive	46	18.9 ± 8.1	20	17.1 ± 7.4	26	20.3 ± 8.4	*n.s.*
Negative	171	17.8 ± 8.2	90	14.7 ± 7.3	81	21.2 ± 7.8	*<0.001*
*p-Value*	*n.s.*		*n.s.*		*n.s.*		
**Tumor Size**							
T0-T1	55	17.8 ± 9.6	35	14.5 ± 7.4	20	23.6 ± 10.3	*<0.001*
T2	98	18.3 ± 8.1	49	16.2 ± 8.3	49	20.6 ± 7.4	*0.007*
T3-T4	56	18.0 ± 7.1	23	15.2 ± 6.1	33	20.0 ± 7.3	*0.013*
*p-Value*	*n.s.*		*n.s.*		*n.s.*		
**Lymph Node**							
Negative	89	18.8 ± 8.9	50	15.8 ± 7.9	39	22.8 ± 8.6	*<0.001*
Positive	120	17.6 ± 7.8	57	15.2 ± 7.3	63	19.9 ± 7.6	*0.001*
*p-Value*	*n.s.*		*n.s.*		*n.s.*		
TNM* Staging							
I/II	146	18.1 ± 8.6	81	15.6 ± 8.1	65	21.2 ± 8.4	*<0.001*
III/V	63	18.2 ± 7.4	26	14.8 ± 5.7	37	20.6 ± 7.6	*0.002*
*p-Value*	*n.s.*		*n.s.*		*n.s.*		
**Subtype**							
ER/PR+/HER2−	96	18.1 ± 8.5	49	15.1 ± 8.6	47	21.2 ± 7.2	*<0.001*
HER2+	46	18.9 ± 8.1	20	17.1 ± 7.4	26	20.3 ± 8.5	*n.s.*
TNBC ^	75	17.4 ± 7.8	41	14.2 ± 5.1	34	21.3 ± 8.7	*<0.001*
*p-Value*	*n.s.*		*n.s.*		*n.s.*		

^ TNBC: ER−/PR−/HER2−; * TNM: TNM Staging System is based on the extent of the tumor (T), the extent of spread to the lymph nodes (N), and the presence of metastasis (M). The T category describes the original (primary) tumor; *“n.s.”: no statistical significance*.

**Table 6:** Association of 25(OH)D3 level and subtypes of breast cancer.

Tumor Subtype	African American			Hispanic		
	OR ^	95% CI	*p*-Value	OR ^	95% CI	*p*-Value
***TNBC type***						
25(OH)D3 level						
>20 ng/mL vs. ≤ 20 ng/mL	5.4	1.4–15	0.02	0.8	0.3–2.2	0.72
**ER/PR−/HER2+ type**						
25(OH)D3 level						
>20 ng/mL vs. ≤ 20 ng/mL	0.4	0.2–2.0	0.25	0.7	0.2–2.5	0.59
***ER/PR+ type***						
25(OH)D3 level						
>20 ng/mL vs. ≤ 20 ng/mL	0.4	0.1–1.1	0.06	0.9	0.4–2.3	0.91

^ adjusted for age at the time of diagnosis, the seasons of blood draw and BMI.

**Table 7:** Cox regression analysis for relative risk of disease-free and overall survival.

Serum 25(OH)D3	Univariate Analysis			Multivariate Analysis ^		
**Disease-free survival**	RR	95% CI	*p-Value*	RR	95% CI	*p-Value*
>24 ng/mL	1			1		
18 ng/mL–23 ng/mL	2.3	1.0–4.8	*0.03*	1.6	0.8–4.8	*0.15*
13 ng/mL–17 ng/mL	2.3	1.1–2.3	*0.02*	2.2	0.9–5.0	*0.07*
≤12 ng/mL	1.7	0.8–3.5	*0.18*	1.9	0.7–3.8	*0.26*
**Overall survival**						
>24 ng/mL	1			1		
18 ng/mL–23 ng/mL	2.8	0.7–4.8	*0.14*	2.4	0.5–11.6	*0.07*
13 ng/mL–17 ng/mL	3.1	0.8–4.9	*0.09*	3.4	0.7–16.1	*0.13*
≤12 ng/mL	2.1	0.5–3.6	*0.31*	4.4	0.9–22.7	*0.28*

RR: Relative risk; ^ adjusted for ethnicity, tumor size, node stage, estrogen receptor, progesterone receptor and HER2 receptor status, BMI, age at the time of diagnosis and season of blood draw.
